# CIPK15-mediated inhibition of NH_4_^+^ transport protects *Arabidopsis* from submergence

**DOI:** 10.1016/j.heliyon.2023.e20235

**Published:** 2023-09-15

**Authors:** Yen-Ning Chen, Cheng-Hsun Ho

**Affiliations:** Agricultural Biotechnology Research Center, Academia Sinica, Taipei, 115, Taiwan

**Keywords:** *Arabidopsis thaliana*, Ammonium, Protein kinase, Phosphorylation, Submergence, Transporter

## Abstract

Ammonium (NH_4_^+^) serves as a vital nitrogen source for plants, but it can turn toxic when it accumulates in excessive amounts. Toxicity is aggravated under hypoxic/anaerobic conditions, e.g., during flooding or submergence, due to a lower assimilation capacity. AMT1; 1 mediates NH_4_^+^ uptake into roots. Under conditions of oxygen-deficiency, i.e., submergence, the CBL-interacting protein kinase OsCIPK15 has been shown to trigger SnRK1A signaling, promoting starch mobilization, thereby the increasing availability of ATP, reduction equivalents and acceptors for NH_4_^+^ assimilation in rice. Our previous study in Arabidopsis demonstrates that AtCIPK15 phosphorylates AMT1; 1 whose activity is under allosteric feedback control by phosphorylation of T460 in the cytosolic C-terminus. Here we show that submergence cause higher NH_4_^+^ accumulation in wild-type, plant but not of nitrate, nor in a quadruple *amt* knock-out mutant. In addition, submergence triggers rapid accumulation of *AtAMT1;1* and *AtCIPK15* transcripts as well as AMT1 phosphorylation. Significantly, *cipk15* knock-out mutants do not exhibit an increase in AMT1 phosphorylation; however, they do display heightened sensitivity to submergence. These findings suggest that CIPK15 suppresses AMT activity, resulting in decreased NH_4_^+^ accumulation during submergence, a period when NH_4_^+^ assimilation capacity may be impaired.

## 1 Introduction

Nitrogen is a fundamental nutrient for all living organisms, as it plays a crucial role in various vital macromolecules, including nucleic acids, amino acids, and proteins. In the context of plants, nitrogen availability can significantly influence growth, development, and crop yield, either by being excessive or insufficient, outside the optimum range. In most soils, nitrogen is mainly present in inorganic forms (as nitrate, NO_3_^−^, and as the ammonium ion, NH_4_^+^). NH_4_^+^ serves as a crucial nitrogen source for bacteria, fungi, and plants. However, it can become toxic when its concentration exceeds certain levels. In many conditions, plants preferentially take up NH_4_^+^ over NO_3_^−^ [[1], [2], [3], [4], [5]]. Average NH_4_^+^ levels in the soil are often 10 to 1000 times lower than NO_3_^−^ levels [2]. NH_4_^+^ availability and distribution vary both spatially and temporally. Elevated NH_4_^+^ levels can be toxic, although the sensitivity to NH4+ varies among different plant species [6]. Soil conditions also affect the nitrogen pool and nitrogen availability.

Under hypoxic conditions, such as flooding or submergence, the conversion of NO_3_^−^ to NH_4_^+^ (nitrification) is blocked, leading to NH_4_^+^ accumulation in the soil [7,8]. At the same time, flooded roots are deprived of oxygen, limiting ATP production and nitrogen assimilation, which then impairs the conversion of NH_4_^+^ to glutamine, aggravating NH_4_^+^ toxicity. Ethylene plays a crucial role in acclimation to flooding which causes hypoxia [9]. Interestingly, ethylene and NH_4_^+^ toxicity appear to be tightly linked, as seen when treatment of tomatoes grown in solution with ethylene inhibitors prevents NH_4_^+^ toxicity [10]. NH_4_^+^ accumulation and ethylene biosynthesis appear to be indicators of stress and may play roles in the development of nutritional stress symptoms. Ethylene evolution is preceded by NH_4_^+^ accumulation but is detected before or at the same time as toxicity symptoms.

Plants utilize specific transporters to uptake NH_4_^+^. The transport of NH_4_^+^ is electrogenic and relies on high-affinity NH_4_^+^ transporters from the AMT/MEP/Rhesus protein superfamily [[11], [12], [13], [14]]. Within the Arabidopsis genome, six AMT paralogs exist, with four of them (AMT1; 1-1; 5) playing an essential role in NH_4_^+^ uptake [15,16]. Besides their function as transporters, AMTs also act as receptors, influencing root growth and development, similar to the yeast MEP2 transceptor that regulates pseudohyphal growth based on NH_4_^+^ concentrations [17,18]. The activity of AMTs is under strict regulation, subject to feedback inhibition to prevent NH_4_^+^ accumulation to toxic levels [19,20]. Previous studies have demonstrated that NH_4_^+^-triggered phosphorylation of a critical threonine (T460) in the cytosolic C-terminus of AMT1; 1 leads to transport inhibition through allosteric regulation within the trimeric transporter complex [21,22]. This C-terminal domain of AMT1; 1, acting as an allosteric switch, is highly conserved among AMT homologs across species, from bacteria to higher plants [12,13,23]. Utilizing this allosteric regulation mechanism of AMT1; 1 for feedback control enables plants to rapidly and efficiently block NH_4_^+^ uptake before reaching toxic levels [21,22]. However, the complete pathway responsible for NH_4_^+^-dependent phosphorylation of AMTs remains incompletely understood. It is speculated that specific kinases may be activated during submergence to restrict NH_4_^+^ accumulation.

In our recent comprehensive investigations using Xenopus oocytes, we have discovered the Arabidopsis protein kinase CIPK15 as a direct interactor that exerts a negative influence on AMT1 activity, thereby serving as an AMT regulator [24]. This inhibitory impact of CIPK15 on AMT1 activity was further validated using an NH4+ transporter activity sensor, AmTryoshka1; 3 LS-F138I, a genetically encoded biosensor with ratiometric capabilities in yeast [25]. It is worth noting that members of the CIPK kinase family are well-known for their ability to regulate the activities of various transporter types [22,[26], [27], [28], [29], [30], [31]]. CIPK15 from rice (also named PKS3/SnRK3.1) has been proposed to be a key regulator of the sensing cascade for successful rice germination under flooding [32]. Under oxygen-deprived conditions, OsCIPK15 triggers both accumulation of the Snf1-related protein kinase 1, SnRK1A, and SnRK1A-dependent signaling to promote starch degradation [[32], [33], [34]]. CIPK15 also appears to play a role in abscisic acid (ABA) signaling, which is also linked to sugar signaling [[35], [36], [37], [38]]. In this study, we demonstrate that *Arabidopsis* CIPK15, a close homolog of OsCIPK15 functions as a negative regulator of AMT1 activity during submergence conditions. The NH_4_^+^ content in Col-0 but not *qko* mutant, which is the quadruple AMT1 transporter mutant, was largely increased under submergence condition. The transcripts of *CIPK15* and *AMT1;1* were largely increased under submergence condition. *cipk15 knock-out* ***mutants*** were hypersensitive to submergence condition as showing the NH_4_^+^ toxicity symptoms. Furthermore, we observed a significant increase in the phosphorylation level of AMT1 under submergence conditions, but this effect was absent in *cipk15* mutants. Collectively, these findings strongly suggest that during submergence, CIPK15 actively suppresses AMT activity to hinder excessive NH_4_^+^ accumulation when the NH_4_^+^ assimilation capacity is compromised.

## 2 STAR methods

**Plant Material and Treatments.***Arabidopsis thaliana* ecotype Columbia-0 was used in this study, and T-DNA insertion lines for CIPK15, namely cipk15-1 (SALK_203,150) and cipk15-2 (GK604B06), were acquired from the Arabidopsis Biological Resource Center (http://www.arabidopsis.org/abrc/). The identification of AMT-*qko*, the quadruple AMT *knock out* line, and *cipk15 knock-out* line has been described [24,39]. Wild-type plants were *Arabidopsis thaliana* ecotype Columbia-0. Plant growth conditions for qRT-PCR analyses and protein blot assay have been described previously, here used with modifications [22] for submergence. *Arabidopsis* seeds underwent surface sterilization and were germinated on 0.5% modified Murashige Skoog medium (MS), nitrogen-free salts (Phytotechlab, M407), with 5 mM KNO_3_ as the exclusive nitrogen source. The medium also contained 0.5% [w/v] sucrose and 1% [w/v] agar, with a pH of 5.8 [KOH], and the plates were kept in a vertical orientation. Seedlings were incubated in a 16-/8-h light/dark period at 22 °C. For submergence experiments, we followed the protocol from the Bailey-Serres's lab [40] with modifications. Briefly, seedlings grown in vertical orientation for 7 d on solid 0.5% MS medium containing 0.5% (w/v) sucrose were submerged under 1 mM NH_4_Cl or 1 mM KNO_3_ medium in a dark, 22 °C climate room. After 13 h of submergence, sample of seedlings or roots was collected after the indicated treatments and frozen in liquid nitrogen for qRT-PCR, protein blot experiments, and NH_4_^+^ level measurements, or seedlings were returned to aerobic conditions on agar plates at pH 6 with 1 mM NH_4_Cl or 1 mM KNO_3_ as the sole nitrogen source to observe post submergence recovery and for NH_4_^+^ level measurements and image scan. Seedlings were scanned on a flatbed scanner.

**Real-Time qRT-PCR analyses.** Real-time qRT-PCR was performed as described [24,27]. In brief, primers were designed to achieve a melting temperature (Tm) of approximately 60 °C and generate PCR products of around 200−400 base pairs. For each experiment, expression levels were initially standardized by comparing them to the expression of Ubiquitin10, measured from the same cDNA samples. The primer sequences used were as follows: *AMT1;1*, forward primer: 5′- ACGACATTATCAGTCGC; reverse primer: 5′-CTGTCCTGTGTAGATTAACG; and *CIPK15*, forward primer: 5′-GGCTACGCATCTGACT; reverse primer: 5′-CGTGCAAGCGACTATC; *Ubiquitin10*, forward primer: 5′-CTTCGTCAAGACTTTGACCG; reverse primer: 5′-CTTCTTAAGCATAACAGAGACGAG.

**Extraction of membrane fractions and protein gel blot analyses**. For plasma membrane extraction, roots were ground in liquid nitrogen and then resuspended in a buffer containing 250 mM Tris-Cl (pH 8.5), 290 mM sucrose, 25 mM EDTA, 5 mM β-mercaptoethanol, 2 mM DTT, 1 mM phenylmethylsulfonyl fluoride (PMSF), 0.53 mM Complete Protease Inhibitor Cocktail (Sigma-Aldrich), and 0.53 mM PhosStop Phosphatase Inhibitor Cocktail (Roche Applied Science). The samples were centrifuged at 10,000×*g* for 15 min, and the resulting supernatants were filtered through Miracloth (Calbiochem) and recentrifuged at 100,000×*g* for 45 min. The microsome-containing sediment was resuspended in a storage buffer consisting of 400 mM mannitol, 10% glycerol, 6 mM MES/Tris (pH 8), 4 mM DTT, 2 mM PMSF, and 13 mM phosphatase inhibitor cocktails 1 and 2 (Sigma-Aldrich). To denature the proteins, they were incubated in loading buffer (62.5 mM Tris-HCl, pH 6.8, 10% [v/v] glycerol, 2% [w/v] SDS, 0.01% [w/v] bromophenol blue, and 1% PMSF) at 37 °C for 30 min, with or without 2.5% [v/v] β-mercaptoethanol at 0 °C. Electrophoresis was then performed using 8–20% SDS polyacrylamide gels (Invitrogen), and the proteins were subsequently transferred to polyvinylidene fluoride membranes. Detection of proteins was accomplished using either the anti-AMT1; 1 antibody or the anti-P-AMT1 T460 antibody [22]. Blots were developed using an ECL Advance Western Blotting Detection Kit (Amersham). Protein and phosphorylation levels were quantified using ImageJ software.

## 3 Results

### 3.1 Submergence causes NH_4_^+^ accumulation

**Fig. 1:**
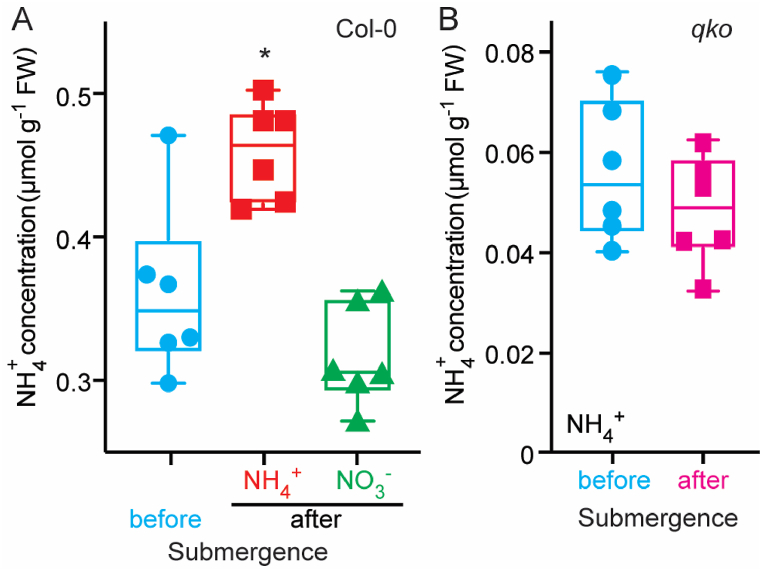
**Submergence increased NH**_**4**_^**+**^**accumulation in Col-0.** NH_4_^+^ concentrations were measured in Col-0 (A) roots under submergence condition in 1 mM NH_4_^+^ or 1 mM NO_3_^−^ and in *qko* (B) roots under submergence in 1 mM NH_4_^+^. Comparable results were obtained in six independent experiments (each experiment n > 12–15, total n > 80). * Indicates *p* < 0.01 compared to Col-0 before submergence (Student's t-test).

NH_4_^+^ accumulation has been observed in a variety of plants. To test whether submergence also causes NH_4_^+^ accumulation in *Arabidopsis,* wild-type Col-0 7-day-old seedlings were submerged under medium in the presence of either NH_4_^+^ or NO_3_^−^ for 13 h in dark. Submergence led to a significant increase in NH_4_^+^ levels in wild-type roots when NH_4_^+^, but not NO_3_^−^, was present (Fig. 1A). To evaluate if NH_4_^+^ accumulation under submergence involved only endogenous accumulation due to reduced assimilation, or if NH_4_^+^ uptake from the medium also played a role, NH_4_^+^ levels in seedlings of a quadruple knock-out AMT mutant (*qko*)*,* which carries T-DNA insertions in *AMT1;1*, *1;2*, *1;3*, and *2;1*, were measured. Submergence did not cause an increase of NH_4_^+^ levels in the *qko* mutant in the NH_4_^+^ condition (Fig. 1B), implicating AMT-mediated NH_4_^+^-uptake in the elevation of NH_4_^+^ levels during submergence.

### 3.2 Submergence and NH_4_^+^-induced *CIPK1*5 mRNA accumulation

**Fig. 2:**
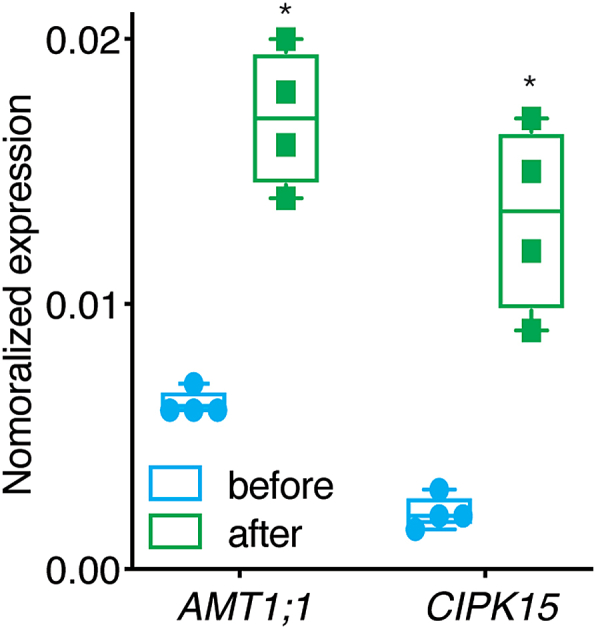
**Submergence-triggered *CIPK1*5 mRNA accumulation.** qRT-PCR analyses of *AMT1;1* and *CIPK1*5 mRNA levels in roots before and after submergence in 1 mM NH_4_^+^. Levels were normalized to *UBQ10* (mean ± SE for four independent experiments. (Each experiment n > 50, total n > 200). * Indicates *p* < 0.01 for mRNA levels of *AMT1;1* and *CIPK15* either after submergence compared to before submergence (Student's t-test).

In roots, CIPK15 governs the NH4+ uptake activity of AMT1; 1 [24]. Based on the role of the rice homolog in hypoxia, we hypothesized that the *Arabidopsis* CIPK15 may also play a role under submergence conditions, and thus affect NH_4_^+^ uptake. During submergence, CIPK15 could undergo regulation at the transcriptional level or activation, which enables it to control AMT activity. Using public database searches (eFP browser) and qRT-PCR analyses, we found that *AMT1;1* transcript levels were elevated in *Arabidopsis* roots and leaves after flooding [36]. (Fig. 2 and *Supplementary* Figs. S1–3). In response to submergence, levels of CIPK15 transcript increased about five-fold in Col-0 and the *qko* mutant (*Supplementary* Fig. S2). The data strongly support the idea that both AMT1; 1 and CIPK15 are involved in the response to submergence.

### 3.3 CIPK15 is a key factor in flooding tolerance in *arabidopsis*

**Fig. 3:**
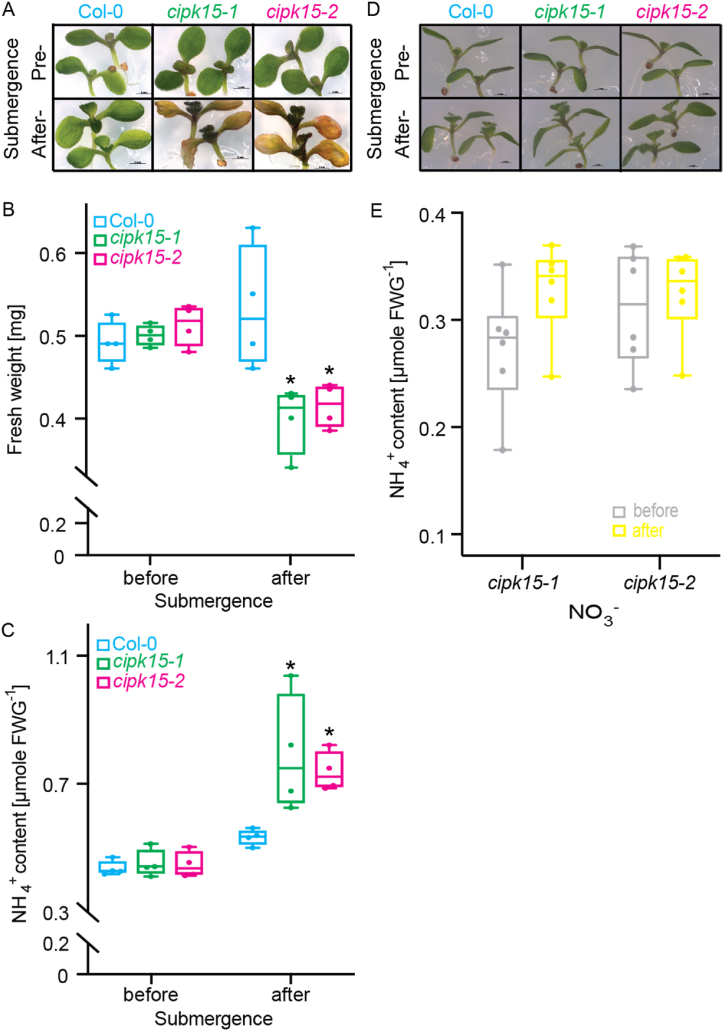
***cipk15* mutants are sensitive to submergence and high NH**_**4**_^**+**^**accumulation under NH**_**4**_**Cl treatment.** Stratified seeds grown in the presence of 0.5% (w/v) sucrose on vertically oriented plates under a diurnal light cycle for 7 d and submerged into water in the present of NH_4_^+^ (A–C) or NO_3_^−^ (D–E) for up to 13 h. (A, D) Representative before (pre-submergence) and after (after-submergence) seedlings following recovery (2 days), (B) fresh weight, and (C, E) NH_4_^+^ concentration in roots of seedlings before and after under submergence in the present of NH_4_^+^ or NO_3_^−^ (each experiment n > 15, total n > 80) in wild-type Col-0 and two *cipk15* knock-out mutants. * Indicates significant change (*p* < 0.01) compared to wild-type (Student's t-test).

We show here that NH_4_^+^ accumulates in seedlings during submergence (Fig. 1). To investigate whether CIPK15 regulates NH_4_^+^ toxicity by modulating AMT1; 1 transport activity during submergence, *cipk15* mutants and wild-type plants were assessed under submerged conditions. Following submergence, *cipk15* mutant seedlings displayed heightened sensitivity to NH_4_^+^ but not NO_3_^−^, and exhibited reduced fresh weight compared to the wild-type (Fig. 3A–B and 3D). Additionally, NH_4_^+^ levels were significantly higher in *cipk15* mutants compared to wild-type controls under NH_4_^+^ conditions, while no such difference was observed under NO_3_^−^ conditions (Fig. 3C and E). These findings collectively suggest that CIPK15 is crucial for restricting NH_4_^+^ accumulation during submergence.

### 3.4 Upon submergence, CIPK15 induces the phosphorylation of AMT1

**Fig. 4:**
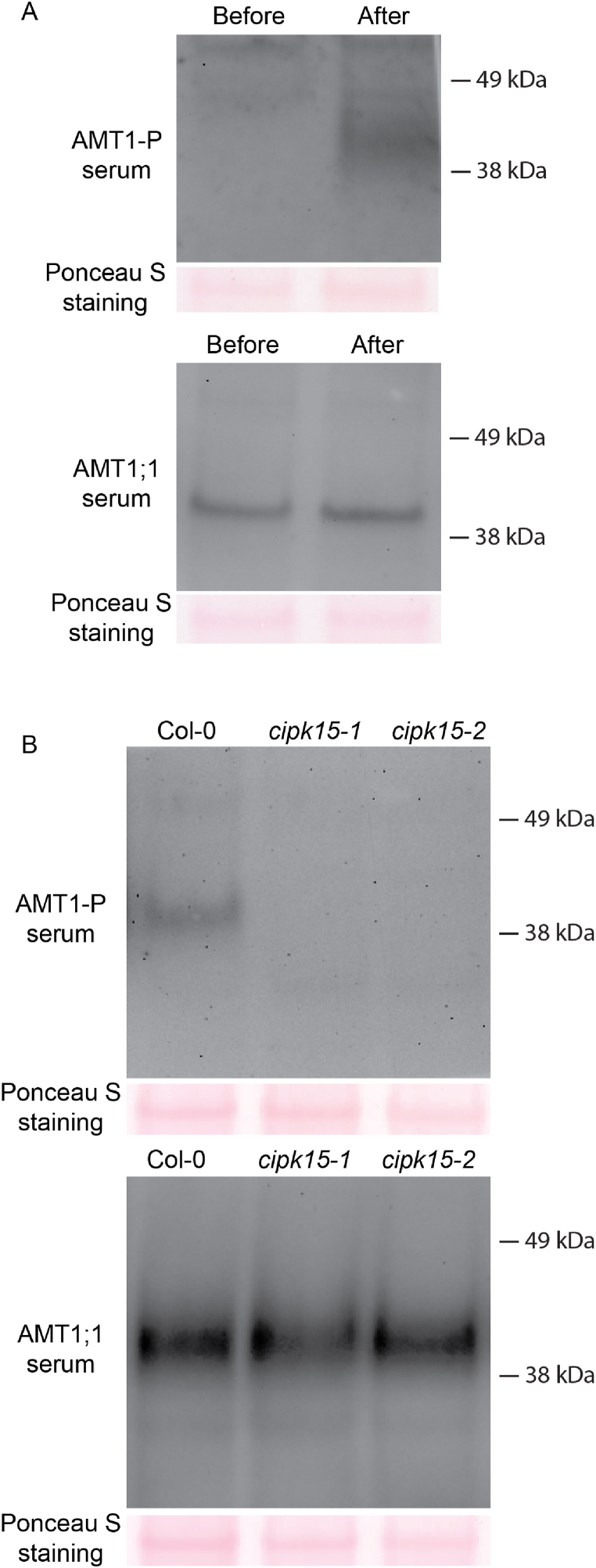
**AMT1;1-T460 phosphorylation is increased under submergence and is reduced in *cipk15* mutant plants.** Before and after submergence of Col-0 seedlings (A) and seedlings after submergence (B) in present of NH_4_^+^, membrane proteins were probed with anti-AMT1; 1 serum and anti-AMT1-P serum [22]. Ponceau S staining serves as the loading control. Corresponding result as shown in Supplementary Fig. S5.

CIPK15 has been demonstrated to interact directly with AMT1; 1, inhibiting its activity through phosphorylation of T460 in the cytosolic C-terminus. To investigate whether the restriction of NH_4_^+^ accumulation during submergence is a result of AMT1; 1 activity inhibition by CIPK15, the phosphorylation status of T460 in AMT1; 1 was examined in both wild-type Col-0 and cipk15 mutant plants. Protein gel blots were incubated with either anti-AMT1; 1 or anti-P-T460 antibody, which recognizes the phosphorylation status of T460 *in vivo* [22]. Following submergence and exposure to NH_4_^+^, the protein levels of AMT1; 1 in both wild-type and *cipk15* mutants remained similar compared to the pre-submergence condition. However, in wild-type Col-0, AMT1; 1 phosphorylation exhibited a significant increase, while this effect was not observed in *cipk15* mutants (Fig. 4A–B). These findings provide strong evidence that submergence triggers CIPK15-mediated phosphorylation of AMT1; 1 at position T460, which subsequently hinders AMT1; 1 activity, preventing the accumulation of NH_4_^+^ to toxic levels.

## 4 Discussion

Our observations reveal that under submergence conditions, the activity of the triple-barreled NH_4_^+^ transporters (AMTs) can be allosterically regulated through phosphorylation by CIPK15. We found evidence that this regulatory system can serve as a safety mechanism to prevent accumulation of NH_4_^+^ to toxic levels under submergence condition. The conserved threonine (T460) in the cytosolic C-terminus of AMTs can undergo phosphorylation by CIPK15, enabling allosteric regulation [22,24,41]. In this regulatory system, some components are yet unknown, including a hypothetical NH_4_^+^ receptor, a protein kinase that phosphorylates AMTs under submergence conditions, and the regulatory network that triggers AMT inactivation in conditions that otherwise would lead to NH_4_^+^ overaccumulation. Here we demonstrated the submergence-triggered inhibition of AMT activity by CIPK15 to prevent overaccumulation of NH_4_^+^ to toxic level. Under submergence condition, we found that NH_4_^+^ content was highly accumulated in Col-0 in the present of NH_4_^+^ but not *qko* mutant or in the present of NO_3_^−^. Following submergence and exposure to NH_4_^+^, the transcript levels of both *AMT1;1* and *CIPK15* were significantly increased. In the presence of NH_4_^+^, *cipk15* knock-out mutants displayed heightened sensitivity to submergence, exhibiting reduced fresh weights and elevated NH_4_^+^ content compared to Col-0, underscoring the essential role of CIPK15 in protecting against NH_4_^+^ toxicity during submergence. 10.13039/100014337Furthermore, as CIPK15 can directly interact with and phosphorylate AMTs [24], its role in regulating AMTs under submergence is further supported. This is evident from the observation that submergence-induced phosphorylation of T460 in AMT1; 1 is impaired in *cipk15* knock-out mutants, emphasizing the involvement of CIPK15 in AMT regulation during submergence.

Bacteria, fungi, and plants that live in soil are exposed to dramatically varying environmental conditions, including drought, rain, and even flooding/submergence, a condition that limits oxygen availability, prevents efficient respiration, and thus reduces ATP and NADH availability. Both ATP and NADH are essential for efficient NH_4_^+^ assimilation. Submergence has also been reported to modify soil structure and nutrient dynamics, leading to crop losses [42,43]. Submergence of plants affects the concentration of nitrogen and nitrogen metabolism to improve survival and recovery from hypoxia by reducing the potential accumulations of toxic products [42,43]. In submerged/flooded conditions or hypoxic soil, one of the main transformations of nitrogen in soil is the accumulation of NH_4_^+^, whereas, almost all the nitrate disappears in short period of time [[44], [45], [46], [47], [48]]. Conversion of NH_4_^+^ to nitrate by nitrifying bacteria in soil or to amino acids in plant cell is inhibited due to lack of oxygen with hypoxia. Cytosolic NH_4_^+^ levels rise during hypoxia, thus similar to elevated NH_4_^+^ conditions, survival under hypoxic condition might also require efficient feedback inhibition of NH_4_^+^ uptake. Here we show that NH_4_^+^ increased in *Arabidopsis* roots during submergence. This concept of preventing toxic amount of NH_4_^+^ accumulation in plant with hypoxia is also supported by the increasing the activity of glutamate synthase, which is the key enzyme to transfer NH_4_^+^ to amino acids [44]. The increased sensitivity of *cipk15* mutants to submergence supports a role of CIPK15 in protection from hypoxia, possibly through the inhibition of AMT activity.

Calcium (Ca^2+^) signals, widely recognized as a key regulatory signal for many processes in plant cells including plant adaptive responses to the environments, in cytosol is involved in triggering the hypoxia response [49]. Hypoxia causes a rapid elevation of the cellular Ca^2+^ concentration in many plants, including maize, rice, wheat, and *Arabidopsis* [50,51]. Moreover, elevated Ca^2+^ levels significantly influence the nitrogen metabolism and acquisition in hypoxia-stressed roots [44,52,53]. Recently, the Ca^2+^-sensing protein kinase OsCIPK15 was shown to play a central role in flooding tolerance in rice. CIPK15 activates the kinase SnRK1A, thereby connecting anaerobic signaling to SnRK1-mediated sugar-signaling. Activation of sugar metabolism would provide more carbon skeletons and more ATP, both of which are limited under hypoxic conditions [32,54,55]. CIPKs are known to affect the activity of many transporters in a reconstituted heterologous system. In our previous study, we found that the *Arabidopsis* CIPK15 is able to interact with and phosphorylate several AMT paralogs and that coexpression of CIPK15 with the AmTryoshka1; 3 LS-F138I biosensor and AMT1; 1 leads to inactivation of both the sensor and transporter. AMT phosphorylation was triggered by exposure to elevated levels of NH_4_^+^ [24]. Here, we showed that *Arabidopsis cipk15* mutants were more sensitive to NH_4_^+^ under submergence condition compared to wild-type, implicating the kinase in general protection from NH_4_^+^ toxicity, and were more sensitive to submergence.

Many interesting questions remain: (i) if CIPK15-mediated AMT phosphorylation requires ethylene or if the cytosolic NH_4_^+^ and/or Ca^2+^ elevation during submergence are sufficient; (ii) if AMTs serve as NH_4_^+^ sensors to help trigger phosphorylation during submergence; and (iii) if AtCIPK15 triggers SnRK activation to simultaneously increase availability of carbon skeletons and ATP to improve NH_4_^+^ assimilation.

Another interesting question is if rice varieties that grow in flooded paddies and use NH_4_^+^ as the predominant form of nitrogen also use CIPK15 for tolerance to submergence and NH_4_^+^ toxicity. In *Arabidopsis*, CIPK15 can interact with CBL1/4 [56], while in rice, CIPK15 can interact with CBLs (CBL3-8) [38,57]. Although *Arabidopsis* CIPK15 is closely related to rice CIPK15, in the same clade [58], whether the two CIPK15 homologs are orthologs or participate in the same biotic and abiotic stress responses or use the same regulatory mechanisms is still unclear and will need to be further addressed. CIPK15 in rice promotes starch mobilization, increases availability of ATP, affects NH_4_^+^ assimilation [32,54,55]. CIPK15 in rice interacts with the ethylene response transcription factor Sub1A in response to flooding in rice [59]. Thus, it will interesting and important to study the sugar catabolism pathway with CIPK15 on the regulation of ammonium assimilation in submergence in the future. CIPK may also be an important component of other regulatory networks by phosphorylating proteins that mediate signal transduction during potassium uptake, pollen germination, and sugar-, hormone-, and/or ROS-signaling [[60], [61], [62]]. It will be interesting to explore the physiological connections between these processes under conditions that lead to NH_4_^+^ accumulation. For instance, increased potassium uptake to counteract the mechanisms of NH_4_^+^ toxicity may lead to reduced NH_4_^+^ uptake and accumulation, thereby preventing the improper binding of NH_4_^+^ in metabolic processes that require potassium [63].

Exploring the interaction between CIPK15 and CIPK23 would be intriguing, especially considering that CIPK23 has also been demonstrated to phosphorylate AMTs [30]. CIPK23 works with the calcium sensor CBL1 to inhibit the transport activities of AMTs. The transcript of CIPK23 was not changed after submergence (Fig. S4). Thus the CBL-CIPK network or even calcium may act upstream to inhibit NH_4_^+^ transport during submergence. It will be interesting to test if CIPK23 also participates in submergence tolerance.

This work provides a new perspective on the role of allosteric feedback regulation of NH_4_^+^ transport during submergence and has demonstrated a new component of the regulatory circuit, the kinase CIPK15 during submergence. It is worth be noted that *AMT1;1* is a NH_4_^+^-induced gene [24], here we showed that the transcript of *AMT1;1* also trigged under submergence; therefore, a different rate of transcription or factor might involve in the regulation of *AMT1;1* transcript in NH_4_^+^ signaling or submergence conditions. It will be interested to study the submergence responses of AMT1 overexpression plants in different environments, e.g., light and time, in the future. A fuller understanding of the response to submergence may provide new ways to engineer crops with higher flooding tolerance.

## Author contribution statement

Yen-Ning Chen: Conceived and designed the experiments; Performed the experiments; Analyzed and interpreted the data; Contributed reagents, materials, analysis tools or data. </p>;

Cheng-Hsun Ho: Conceived and designed the experiments; Performed the experiments; Analyzed and interpreted the data; Contributed reagents, materials, analysis tools or data; Wrote the paper. </p>

## Data availability statement

Data included in article/supp. material/referenced in article.

## Declaration of competing interest

The authors declare that they have no known competing financial interests or personal relationships that could have appeared to influence the work reported in this paper.

## References

[1] Gazzarrini S., Lejay L., Gojon A., Ninnemann O., Frommer W.B., von Wiren N. (1999). Three functional transporters for constitutive, diurnally regulated, and starvation-induced uptake of ammonium into Arabidopsis roots. Plant Cell.

[2] Marschner H. (1996).

[3] Glass A.D.M., Siddiqi M.Y., Srivastava H.S., Singh R.P. (1995). Nitrogen Nutrition in Higher Plants.

[4] Gojon A., Soussana J.F., Passama L., Robin P. (1986). Nitrate reduction in roots and shoots of barley (Hordeum vulgare L.) and corn (Zea mays L.) seedlings: I. N Study. Plant Physiol..

[5] Fukao T., Xu K., Ronald P.C., Bailey-Serres J. (2006). A variable cluster of ethylene response factor-like genes regulates metabolic and developmental acclimation responses to submergence in rice. Plant Cell.

[6] Britto D.T., Siddiqi M.Y., Glass A.D., Kronzucker H.J. (2001). Futile transmembrane NH_4_^+^ cycling: a cellular hypothesis to explain ammonium toxicity in plants. Proc. Natl. Acad. Sci. U.S.A..

[7] Leyshon A.J., Sheard R.W. (1974). Influence of short-term flooding on the growth and plant nutrient composition of barley. Can. J. Soil Sci..

[8] Drew M.C. (1997). Oxygen deficiency and root metabolism: injury and acclimation under hypoxia and anoxia. Annu. Rev. Plant Physiol. Plant Mol. Biol..

[9] Sasidharan R., Voesenek L.A. (2015). Ethylene-mediated acclimations to flooding stress. Plant Physiol..

[10] Barker A.V., Corey K.A. (1991). Interrelations of ammonium toxicityand ethylene action in tomato. Hortscience.

[11] Ludewig U., von Wiren N., Frommer W.B. (2002). Uniport of NH_4_^+^ by the root hair plasma membrane ammonium transporter LeAMT1;1. J. Biol. Chem..

[12] Marini A.M., Vissers S., Urrestarazu A., Andre B. (1994). Cloning and expression of the MEP1 gene encoding an ammonium transporter in Saccharomyces cerevisiae. EMBO J..

[13] Ninnemann O., Jauniaux J.C., Frommer W.B. (1994). Identification of a high affinity NH_4_^+^ transporter from plants. EMBO J..

[14] Tremblay P.L., Hallenbeck P.C. (2009). Of blood, brains and bacteria, the Amt/Rh transporter family: emerging role of Amt as a unique microbial sensor. Mol. Microbiol..

[15] Duan F., Giehl R.F.H., Geldner N., Salt D.E., von Wiren N. (2018). Root zone-specific localization of AMTs determines ammonium transport pathways and nitrogen allocation to shoots. PLoS Biol..

[16] Yuan L., Loqué D., Kojima S., Rauch S., Ishiyama K., Inoue E., Takahashi H., von Wirén N. (2007). The organization of high-affinity ammonium uptake in Arabidopsis roots depends on the spatial arrangement and biochemical properties of AMT1-type transporters. Plant Cell.

[17] Boeckstaens M., André B., Marini A.M. (2007). The yeast ammonium transport protein Mep2 and its positive regulator, the Npr1 kinase, play an important role in normal and pseudohyphal growth on various nitrogen media through retrieval of excreted ammonium. Mol. Microbiol..

[18] Lima J.E., Kojima S., Takahashi H., von Wiren N. (2010). Ammonium triggers lateral root branching in Arabidopsis in an AMMONIUM TRANSPORTER1;3-dependent manner. Plant Cell.

[19] Wang M.Y., Siddiqi M.Y., Ruth T.J., Glass A. (1993). Ammonium uptake by rice roots (II. kinetics of ^13^NH_4_^+^ influx across the plasmalemma). Plant Physiol..

[20] Kronzucker H.J., Britto D.T., Davenport R.J., Tester M. (2001). Ammonium toxicity and the real cost of transport. Trends Plant Sci..

[21] Loqué D., Lalonde S., Looger L.L., von Wirén N., Frommer W.B. (2007). A cytosolic trans-activation domain essential for ammonium uptake. Nature.

[22] Lanquar V., Loque D., Hormann F., Yuan L., Bohner A., Engelsberger W.R., Lalonde S., Schulze W.X., von Wiren N., Frommer W.B. (2009). Feedback inhibition of ammonium uptake by a phospho-dependent allosteric mechanism in Arabidopsis. Plant Cell.

[23] Tremblay P.L., Hallenbeck P.C. (2009). Of blood, brains and bacteria, the Amt/Rh transporter family: emerging role of Amt as a unique microbial sensor. Mol. Microbiol..

[24] Chen H.Y., Chen Y.N., Wang H.Y., Liu Z.T., Frommer W.B., Ho C.H. (2020). Feedback inhibition of AMT1 NH_4_^+^-transporters mediated by CIPK15 kinase. BMC Biol..

[25] Ast C., Foret J., Oltrogge L.M., De Michele R., Kleist T.J., Ho C.H., Frommer W.B. (2017). Ratiometric Matryoshka biosensors from a nested cassette of green- and orange-emitting fluorescent proteins. Nat. Commun..

[26] Neuhauser B., Dynowski M., Mayer M., Ludewig U. (2007). Regulation of NH_4_^+^ transport by essential cross talk between AMT monomers through the carboxyl tails. Plant Physiol..

[27] Ho C.H., Lin S.H., Hu H.C., Tsay Y.F. (2009). CHL1 functions as a nitrate sensor in plants. Cell.

[28] Lan W.Z., Lee S.C., Che Y.F., Jiang Y.Q., Luan S. (2011). Mechanistic analysis of AKT1 regulation by the CBL-CIPK-PP2CA interactions. Mol. Plant.

[29] Xu J., Li H.D., Chen L.Q., Wang Y., Liu L.L., He L., Wu W.H. (2006). A protein kinase, interacting with two calcineurin B-like proteins, regulates K^+^ transporter AKT1 in Arabidopsis. Cell.

[30] Straub T., Ludewig U., Neuhauser B. (2017). The kinase CIPK23 inhibits ammonium transport in Arabidopsis thaliana. Plant Cell.

[31] Dubeaux G., Neveu J., Zelazny E., Vert G. (2018). Metal sensing by the IRT1 transporter-receptor orchestrates its own degradation and plant metal nutrition. Mol. Cell.

[32] Lee K.-W., Chen P.-W., Lu C.-A., Chen S., Ho T.-H.D., Yu S.-M. (2009). Coordinated responses to oxygen and sugar deficiency allow rice seedlings to tolerate flooding. Sci. Signal..

[33] Lu C.-A., Ho T-hD., Ho S.-L., Yu S.-M. (2002). Three novel MYB proteins with one DNA binding repeat mediate sugar and hormone regulation of α-amylase gene expression. Plant Cell.

[34] Lu C.-A., Lin C.-C., Lee K.-W., Chen J.-L., Huang L.-F., Ho S.-L., Liu H.-J., Hsing Y.-I., Yu S.-M. (2007). The SnRK1A protein kinase plays a key role in sugar signaling during germination and seedling growth of rice. Plant Cell.

[35] Halfter U., Ishitani M., Zhu J.K. (2000). The Arabidopsis SOS2 protein kinase physically interacts with and is activated by the calcium-binding protein SOS3. Proc. Natl. Acad. Sci. U.S.A..

[36] Guo Y., Xiong L., Song C.P., Gong D., Halfter U., Zhu J.K. (2002). A calcium sensor and its interacting protein kinase are global regulators of abscisic acid signaling in Arabidopsis. Dev. Cell.

[37] Song C.P., Agarwal M., Ohta M., Guo Y., Halfter U., Wang P., Zhu J.K. (2005). Role of an Arabidopsis AP2/EREBP-type transcriptional repressor in abscisic acid and drought stress responses. Plant Cell.

[38] Kanwar P., Sanyal S.K., Tokas I., Yadav A.K., Pandey A., Kapoor S., Pandey G.K. (2014). Comprehensive structural, interaction and expression analysis of CBL and CIPK complement during abiotic stresses and development in rice. Cell Calcium.

[39] Cheong Y.H., Pandey G.K., Grant J.J., Batistic O., Li L., Kim B.G., Lee S.C., Kudla J., Luan S. (2007). Two calcineurin B-like calcium sensors, interacting with protein kinase CIPK23, regulate leaf transpiration and root potassium uptake in Arabidopsis. Plant J..

[40] Branco-Price C., Kawaguchi R., Ferreira R.B., Bailey-Serres J. (2005). Genome-wide analysis of transcript abundance and translation in Arabidopsis seedlings subjected to oxygen deprivation. Ann. Bot..

[41] Loqué D., Mora S.I., Andrade S.L., Pantoja O., Frommer W.B. (2009). Pore mutations in ammonium transporter AMT1 with increased electrogenic ammonium transport activity. J. Biol. Chem..

[42] Leyshon A.J., Sheard R.W. (1974). Influence of short-term flooding on growth and plant nutrient composition of barley. Can. J. Soil Sci..

[43] Bacanamwo M., Purcell L.C. (1999). Soybean dry matter and N accumulation responses to flooding stress, N sources and hypoxia. J. Exp. Bot..

[44] Gao H.B., Jia Y.X., Guo S.R., Lv G.Y., Wang T., Juan L. (2011). Exogenous calcium affects nitrogen metabolism in root-zone hypoxia-stressed muskmelon roots and enhances short-term hypoxia tolerance. J. Plant Physiol..

[45] Ponnamperuma F.N. (1972). The chemistry of submerged soils. Adv. Agron..

[46] Koschorreck M., Darwich A. (2003). Nitrogen dynamics in seasonally flooded soils in the Amazon floodplain. Wetl. Ecol. Manag..

[47] Satti P., Mazzarino M.J., Gobbi M., Funes F., Roselli L., Fernandez H. (2003). Soil N dynamics in relation to leaf litter quality and soil fertility in north-western Patagonian forests. J Eco.

[48] Alaoui-Sosse B., Gerard B., Biner P., Toussant M.L., Badot P.M. (2005). Influence of flooding on growth, nitrogen availability in soil, and nitrate reduction of young oak seedlings (Quercus robur L.). Ann Forest Sci.

[49] Aurisano N., Bertani A., Reggiani R. (1995). Involvement of calcium and calmodulin in protein and amino acid metabolism in rice roots under anoxia. Plant Cell Physiol..

[50] Yemelyanov V.V., Shishova M.F., Chirkova T.V., Lindberg S.M. (2011). Anoxia-induced elevation of cytosolic Ca^2+^ concentration depends on different Ca^2+^ sources in rice and wheat protoplasts. Planta.

[51] Subbaiah C.C., Zhang J.K., Sachs M.M. (1994). Involvement of intracellular calcium in anaerobic gene-expression and survival of maize seedlings. Plant Physiol..

[52] Bailey-Serres J., Voesenek L.A.C.J. (2008). Flooding stress: acclimations and genetic diversity. Annu. Rev. Plant Biol..

[53] Shabala S., Shabala L., Barcelo J., Poschenrieder C. (2014). Membrane transporters mediating root signalling and adaptive responses to oxygen deprivation and soil flooding. Plant Cell Environ..

[54] Kudahettige N.P., Pucciariello C., Parlanti S., Alpi A., Perata P. (2011). Regulatory interplay of the Sub1A and CIPK15 pathways in the regulation of alpha-amylase production in flooded rice plants. Plant Biol.

[55] Yim H.K., Lim M.N., Lee S.E., Lim J., Lee Y., Hwang Y.S. (2012). Hexokinase-mediated sugar signaling controls expression of the calcineurin B-like interacting protein kinase 15 gene and is perturbed by oxidative phosphorylation inhibition. J. Plant Physiol..

[56] Guo Y., Halfter U., Ishitani M., Zhu J.K. (2001). Molecular characterization of functional domains in the protein kinase SOS2 that is required for plant salt tolerance. Plant Cell.

[57] Kurusu T., Hamada J., Nokajima H., Kitagawa Y., Kiyoduka M., Takahashi A., Hanamata S., Ohno R., Hayashi T., Okada K. (2010). Regulation of microbe-associated molecular pattern-induced hypersensitive cell death, phytoalexin production, and defense gene expression by calcineurin B-like protein-interacting protein kinases, OsCIPK14/15, in rice cultured cells. Plant Physiol..

[58] Hu W., Xia Z.Q., Yan Y., Ding Z.H., Tie W.W., Wang L.Z., Zou M.L., Wei Y.X., Lu C., Hou X.W. (2015). Genome-wide gene phylogeny of CIPK family in cassava and expression analysis of partial drought-induced genes. Front. Plant Sci..

[59] Bailey-Serres J., Voesenek L.A. (2010). Life in the balance: a signaling network controlling survival of flooding. Curr. Opin. Plant Biol..

[60] Mao J., Manik S.M., Shi S., Chao J., Jin Y., Wang Q., Liu H. (2016). Mechanisms and physiological roles of the CBL-CIPK networking system in Arabidopsis thaliana. Genes.

[61] Weinl S., Kudla J. (2009). The CBL-CIPK Ca^2+^-decoding signaling network: function and perspectives. New Phytol..

[62] Sanyal S.K., Rao S., Mishra L.K., Sharma M., Pandey G.K. (2016). Plant stress responses mediated by CBL-CIPK phosphorylation network. Enzymes.

[63] ten Hoopen F., Cuin T.A., Pedas P., Hegelund J.N., Shabala S., Schjoerring J.K., Jahn T.P. (2010). Competition between uptake of ammonium and potassium in barley and Arabidopsis roots: molecular mechanisms and physiological consequences. J. Exp. Bot..

