# Supplementary material for: CIPK15-mediated inhibition of NH4+ transport protects Arabidopsis from submergence
Source: Heliyon. 2023 Sep 15;9(9):e20235. doi: 10.1016/j.heliyon.2023.e20235 (PMC10560025; doi:10.1016/j.heliyon.2023.e20235)
Supplement: Multimedia component 1 [file mmc1.pdf]

Title:

CIPK15-mediated inhibition of NH_4^+ transport protects *Arabidopsis* from submergence

Yi Ning Chen[1] and Cheng-Hsun Ho[1,*]

Affiliations:

[1] Agricultural Biotechnology Research Center, Academia Sinica, Taipei, 115, Taiwan

*Correspondence should be addressed to C.-H. H. (email: zcybele3@sinica.edu.tw; Tel:+886-2-27872123)

Supplementary file (Supporting information figures)

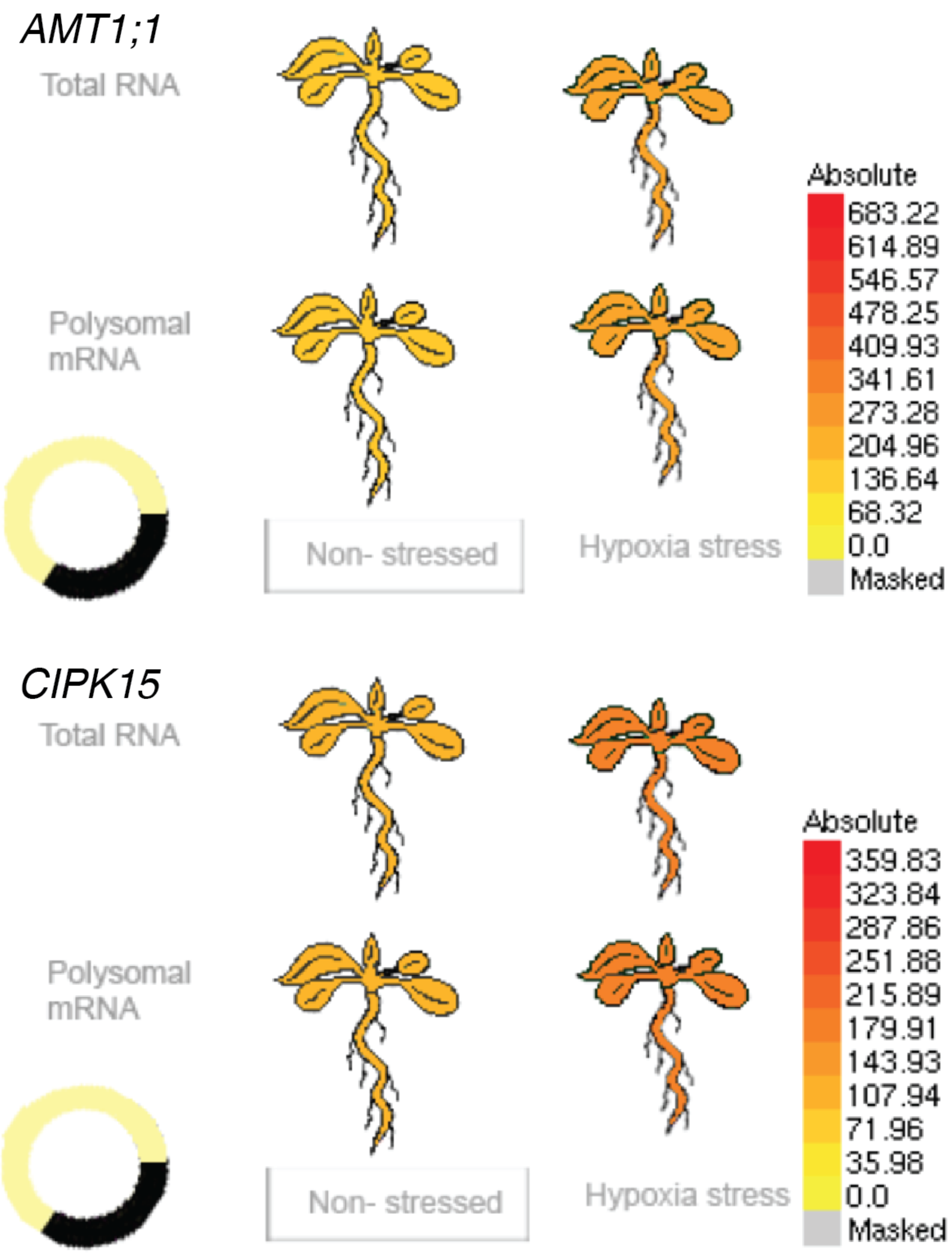

Supporting Information Figure S1. mRNA levels and polysome-associated mRNAs for *CIPK15* and *AMT1;1* increased during hypoxia. *Arabidopsis* eFP browser (efp.ucr.edu) (Branco-Price *et al.*, 2005).

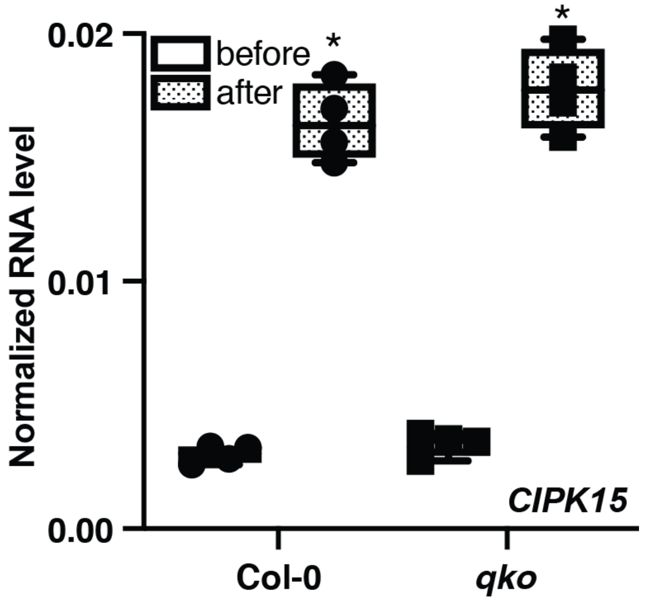

Supporting Information Figure S2. ***CIPK15*** **mRNA levels in Col-0** ***and qko*** **mutant under submergence.** qRT-PCR analyses of *CIPK15* mRNA levels in roots of Col-0 and the quadruple AMT *qko* mutant before and after submergence. *CIPK15* mRNA levels were normalized to *UBQ10* (mean ± SE for four independent experiments. (each experiment n >50, total n >200). An * indicates p <0.05 for mRNA levels of *CIPK15* after submergence compared to before submergence (Student's t-test).

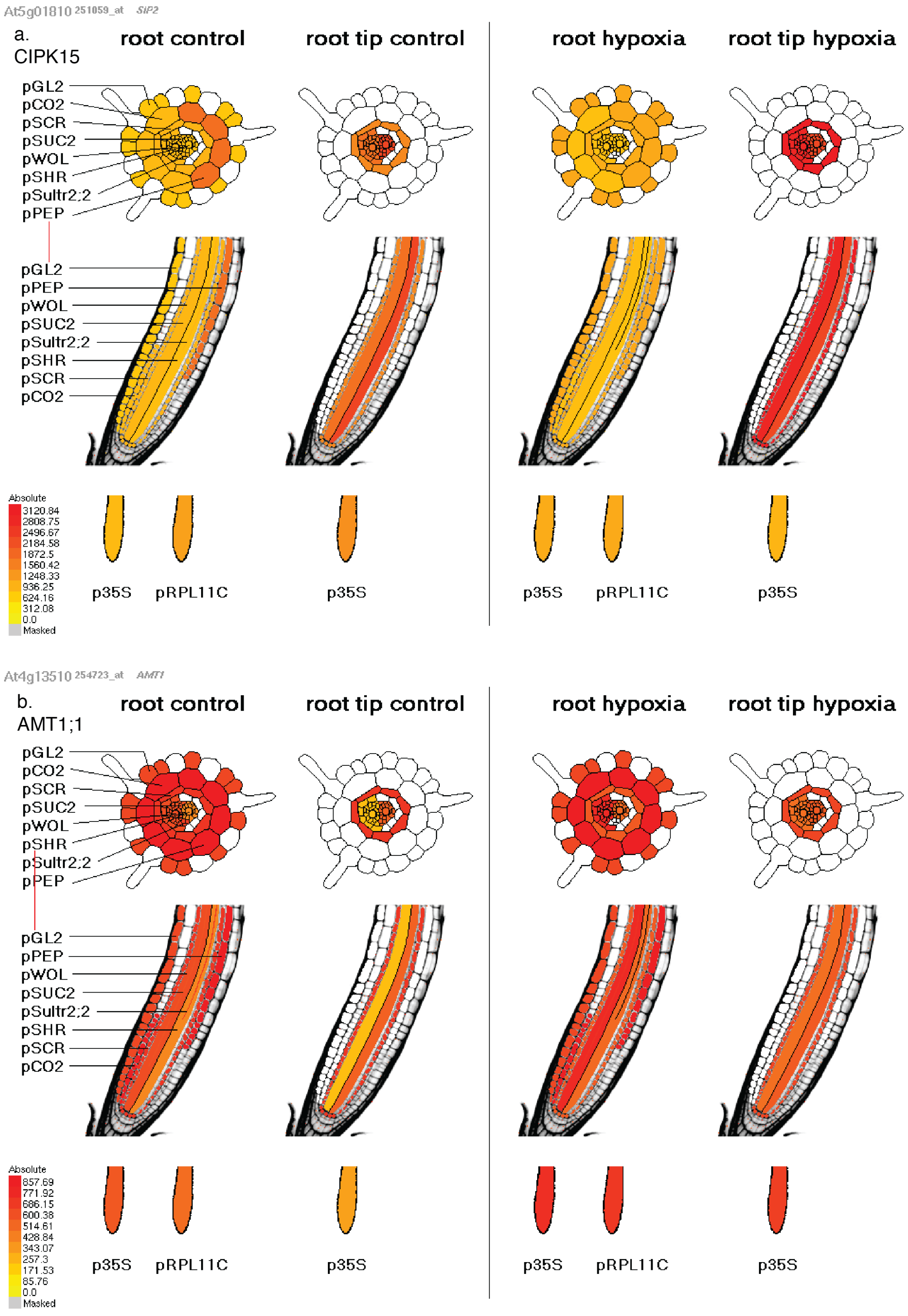

Supporting Information Figure S3. CIPK15 and AMT1;1 are both induced and expressed in the same cells during hypoxia. The root profiling translatomes of CIPK15 (**a**) and AMT1;1 (**b**) during hypoxia in *Arabidopsis* by Julia Bailey-Serres's lab (http://efp.ucr.edu/) (Mustroph *et al.*, 2009).

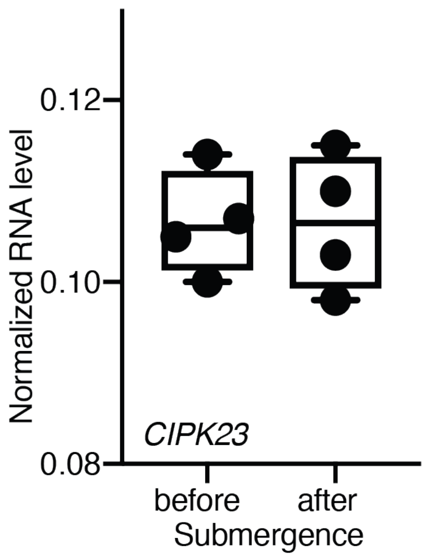

Supporting Information Figure S4. ***CIPK23*** **mRNA levels in Col-0 under submergence.** qRT-PCR analyses of *CIPK23* mRNA levels in roots of Col-0 before and after submergence. *CIPK23* mRNA levels were normalized to *UBQ10* (mean ± SE for four independent experiments. (each experiment n >50, total n >200). No significant different between the mRNA levels before or after submergence (Student's t-test).

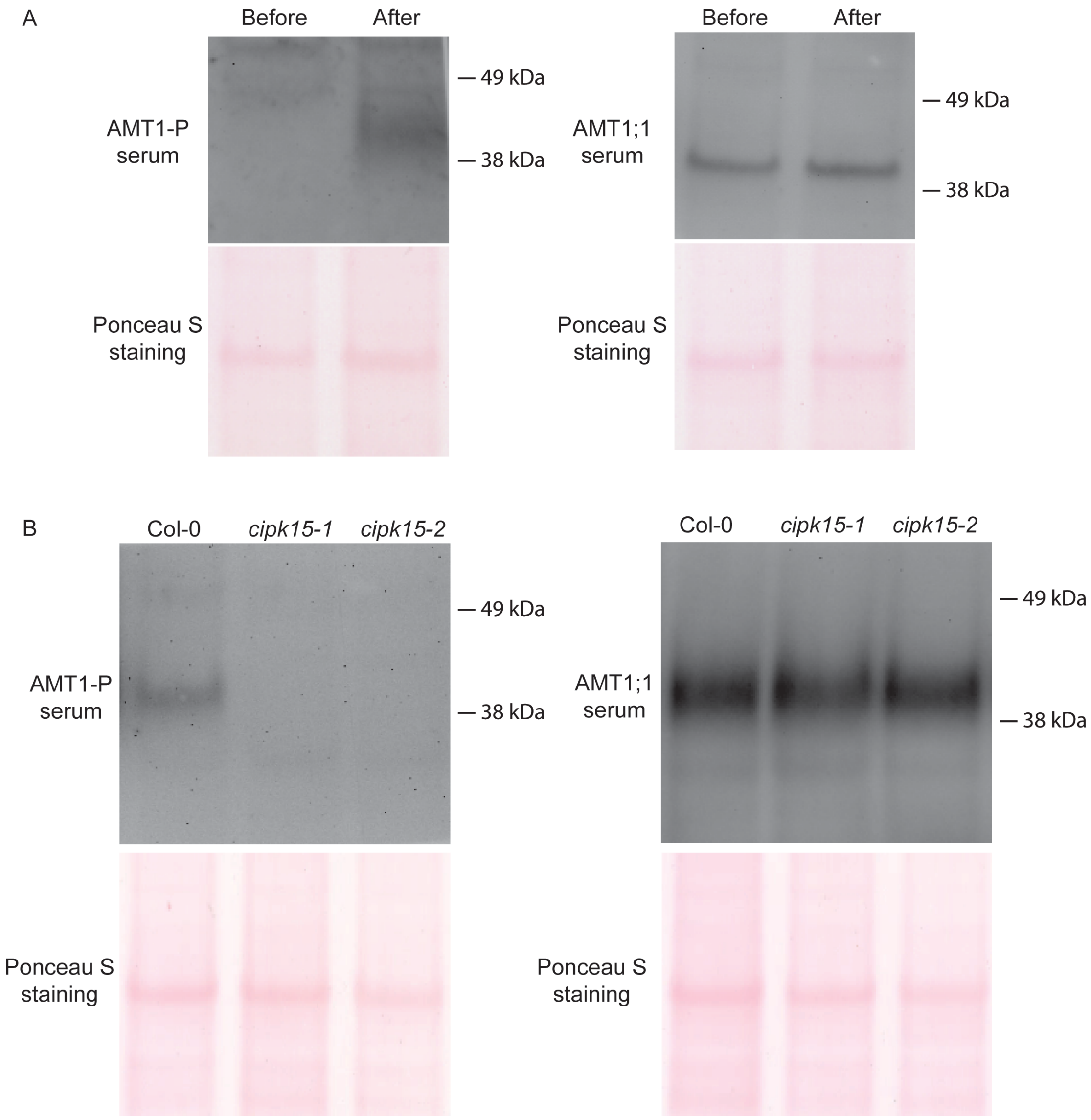

Supporting Information Figure S5. Protein blot in wild type and *cipk15* mutans under submergence. The corresponding result of Figure 4 in main text. Before and after submergence of Col-0 seedlings (A) and seedlings after submergence (B) in present of NH_4^+, membrane proteins were probed with anti-AMT1;1 serum and anti-AMT1-P serum [22]. Ponceau S staining serves as the loading control.
